# Supplementary material for: Optogenetic control of mRNA condensation reveals an intimate link between condensate material properties and functions
Source: Nat Commun. 2024 Apr 15;15:3216. doi: 10.1038/s41467-024-47442-x (PMC11018775; doi:10.1038/s41467-024-47442-x)
Supplement: Supplementary file 1 — Supplementary Information [file 41467_2024_47442_MOESM1_ESM.pdf]

Supplementary Information

Optogenetic control of mRNA condensation reveals an intimate link between condensate material properties and functions

Min Lee[1#], Hyungseok C. Moon[2#], Hyeonjeong Jeong[2,3], Dong Wook Kim[2], Hye Yoon Park[2,3*], Yongdae Shin[1,4*]

[1]Interdisciplinary Program in Bioengineering, Seoul National University, Seoul 08826, Korea
[2]Department of Physics and Astronomy, Seoul National University, Seoul 08826, Korea
[3]Department of Electrical and Computer Engineering, University of Minnesota, Minneapolis 55455, USA
[4]Department of Mechanical Engineering, Seoul National University, Seoul 08826, Korea
[#]These authors contributed equally: Min Lee, Hyungseok C. Moon
*Correspondence: ydshin@snu.ac.kr, hyp@umn.edu

This file includes:

Supplementary Fig. 1 to 9 and figure legends

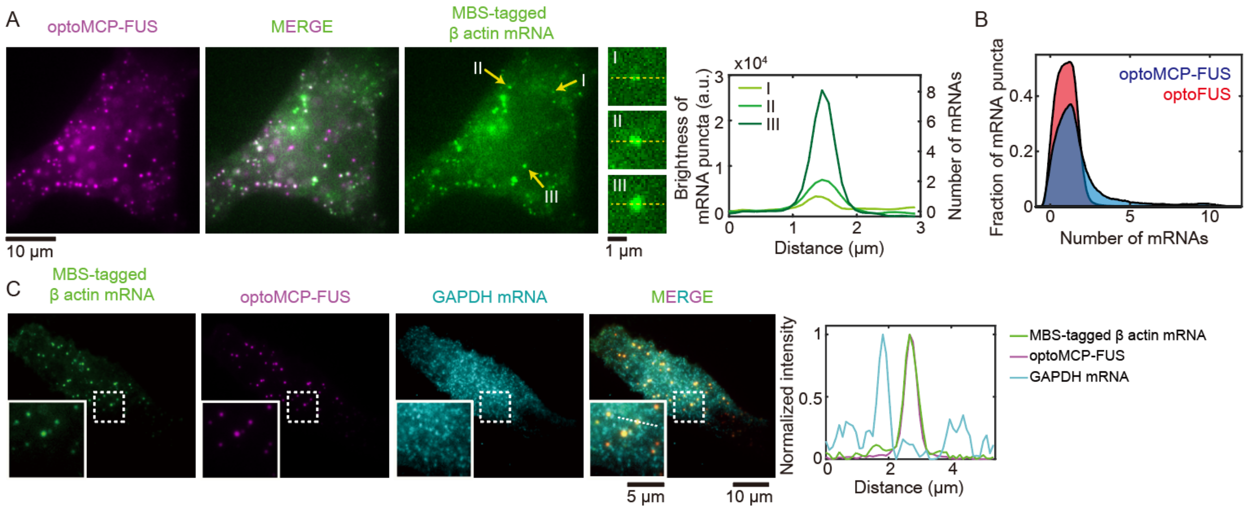

Supplementary Fig. 1: Specific recruitment of the target mRNA into optoMCP-FUS condensates

(A) (Left) Two-color fluorescence images of the fixed optoMCP-FUS expressing MEF cells with the MBS tagged β-actin gene. The MBS tagged β-actin mRNAs, visualized with smFISH (green), and light-activated condensates (magenta) are shown. Examples of mRNA puncta are highlighted with arrows, and their zoomed-in images are shown on the right. (Right) Intensity profile plots along yellow dashed lines in the zoomed-in images. To obtain the number of mRNAs in individual mRNA puncta, fluorescence intensities of mRNA puncta were normalized with the average brightness of a single mRNA in MEF cells expressing optoFUS control. (B) Histogram of the number of mRNAs in individual mRNA puncta in MEF cells expressing optoMCP-FUS or optoFUS. After 20 min of blue light activation, the sample preparation for smFISH was conducted to quantify mRNA recruitment into light-activated condensates. n = 1558 (optoMCP-FUS) and 2580 (optoFUS). (C) Fluorescence images of a fixed optoMCP-FUS expressing MEF cell with the MBS tagged β-actin gene. The cell was exposed to blue light for 20 min to induce phase separation, and then fixed for smFISH. (Left) Three-color images of the MBS-tagged β-actin mRNA (green), optoMCP-FUS (magenta) and GAPDH (blue). (Right) Intensity profile plots for normalized fluorescence intensities along a white dashed line. Source data for panels A, B and C are provided in the Source Data file.

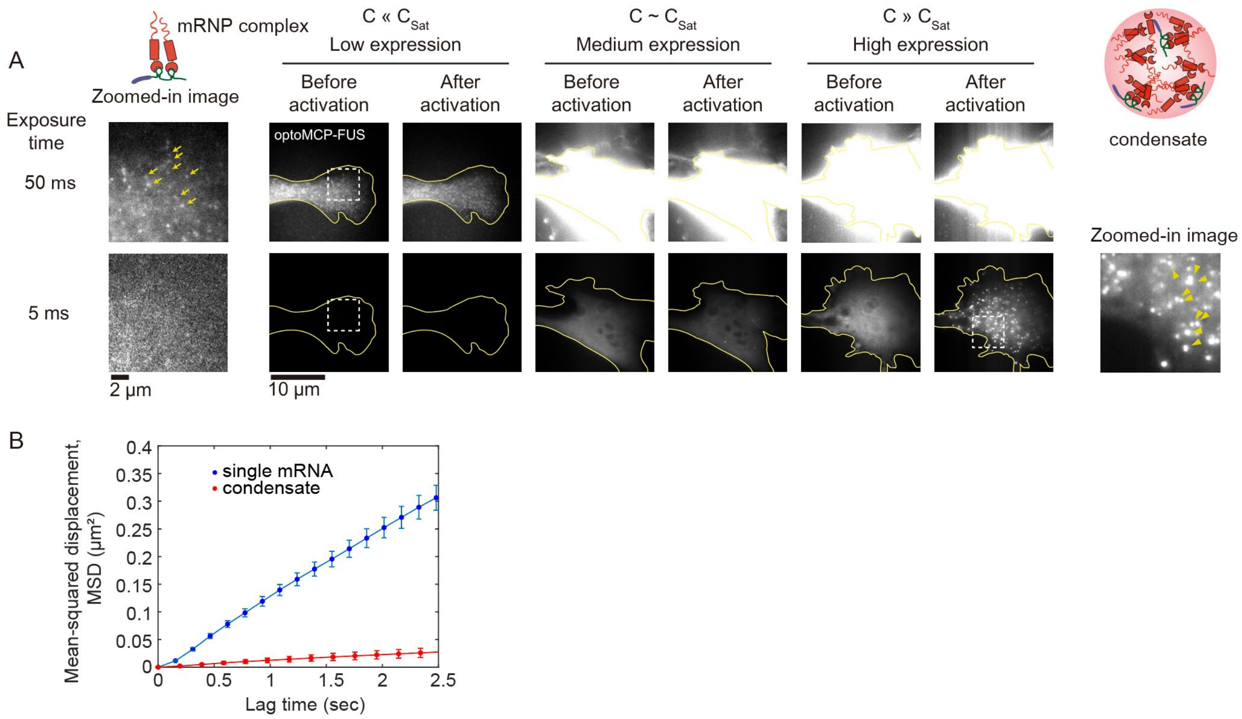

Supplementary Fig. 2: Individual mRNA-optoMCP-FUS complexes are observable at the expression levels below the saturation concentration of optoMCP-FUS

(A) Fluorescence images of the live MEF cell expressing different concentrations of optoMCP-FUS before and after blue-light activation. Images in each row were taken with different EMCCD exposure times, and walking-averaged. The cells were activated with blue light every 10 seconds for 3 minutes. Arrows indicate individual mRNA-optoMCP-FUS complexes, and arrowheads indicate optoMCP-FUS condensates. (B) Average mean-squared-displacement (MSD; ± s.e.m.) of mRNAs and optoMCP-FUS condensates (fitted diffusion constant (D_{mRNA}) = 0.037 ± 0.002 $\mu m^2 s^{-1}$ and ($D_{condensate}$)= 0.0034 ± 0.0013 $\mu m^2 s^{-1}$). n = 24 and 7, respectively. Source data for panel B are provided in the Source Data file.

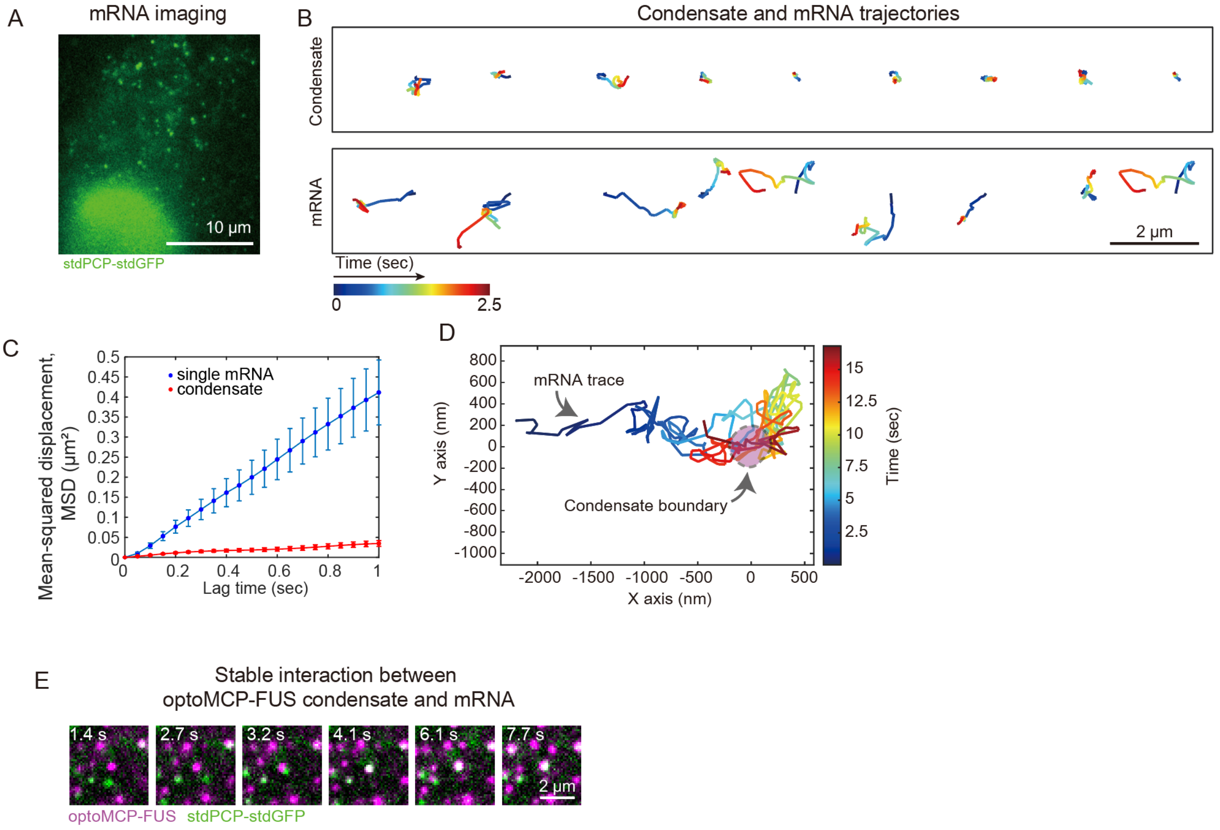

Supplementary Fig. 3: Orthogonal imaging of single mRNAs during light-activated condensation

(A) Fluorescence image of a live immortalized stdPCP-stdGFP expressing MEF cell with endogenous c-FOS gene tagged with MBS-PBS repeats. (B) The 2D trajectories of optoMCP-FUS condensates and the tagged c-FOS mRNAs. Trajectories were acquired from MEF cells expressing opMCP-FUS and stdPCP-stdGFP, and harboring the MBS-PBS-tagged c-FOS gene. (C) Average mean-squared-displacement (MSD; ± s.e.m.) of mRNAs and optoMCP-FUS condensates (fitted diffusion constant (D_{mRNA}) = 0.118 ± 0.023 $\mu m^2 s^{-1}$and ($D_{condensate}$)= 0.014 ± 0.0017 $\mu m^2 s^{-1}$). n = 9 for both. Cells were imaged every 50 ms. (D) 2D trajectory of a tagged c-Fos mRNA interacting with the optoMCP-FUS condensate. (E) Example images of the tagged c-Fos mRNA stably associating with the optoMCP-FUS condensate. Images were walking-averaged and bleach-and-drift-corrected. The MEF cell is imaged every 50 ms after 30 seconds of blue light activation. Source data for panel B are provided in the Source Data file.

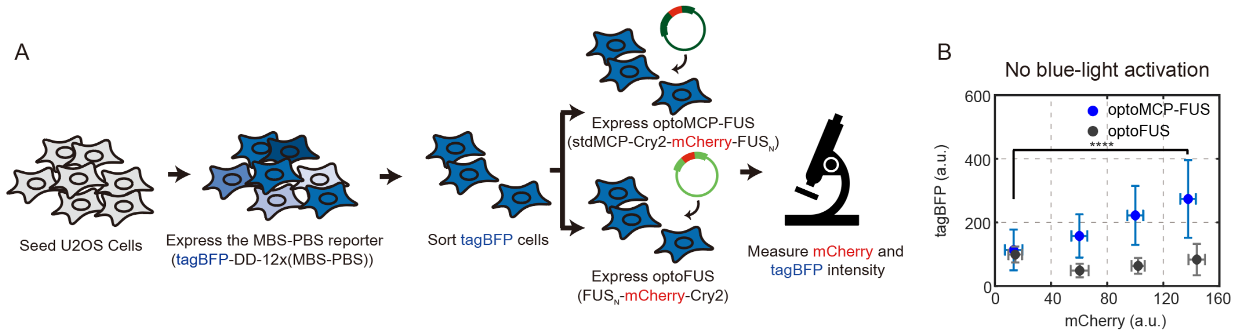

Supplementary Fig. 4: Binding of optoMCP-FUS to the mRNA leads to higher protein expression

(A) U2OS cells expressing the MBS-PBS reporter were sorted to obtain cells with relatively small variation in the tagBFP expression. After transduction of either optoMCP-FUS or optoFUS, mCherry and tagBFP signals in individual cells were measured through the confocal microscope in the absence of any blue light activation. Cells were imaged 2 days after transduction. (B) TagBFP intensity without blue light activation as a function of optoMCP-FUS or optoFUS expression levels. Data are mean ± SD. n = 73 (0-40), 38 (40-80), 24 (80-120), and 30 cells (120-160) for optoMCP-FUS. n = 85 (0-40), 27 (40-80), 20 (80-120) and 15 cells (120-160) for optoFUS. ****$p < 0.001$. p = 3.21E-5. Source data for panel B are provided in the Source Data file.

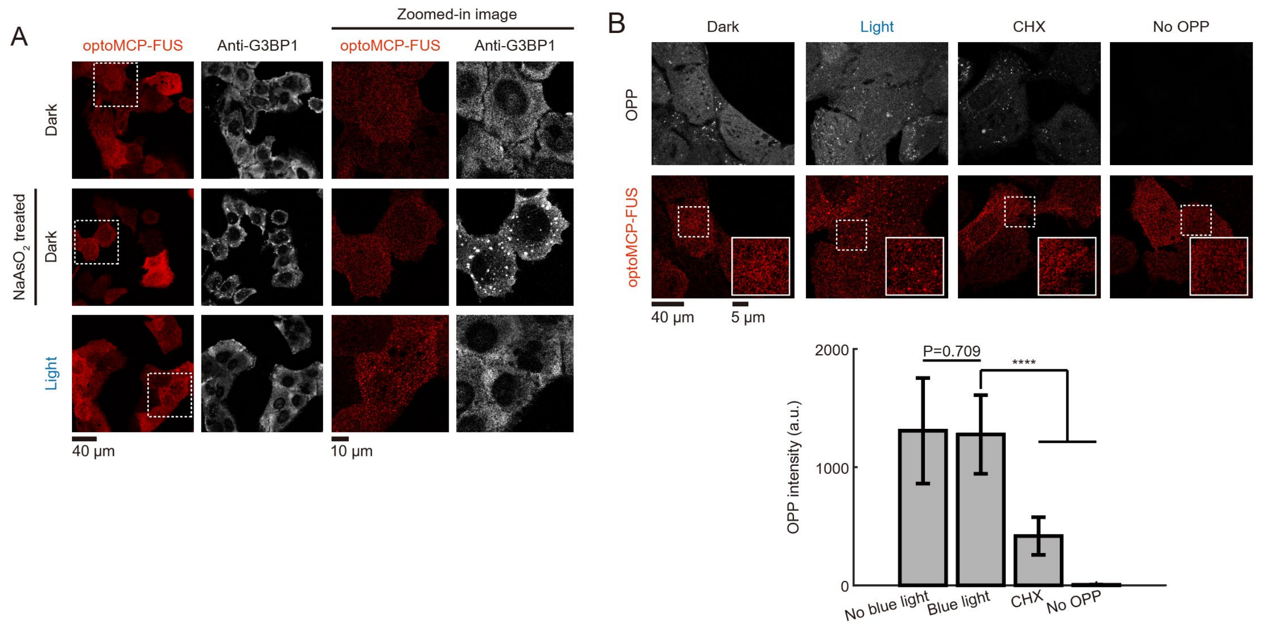

Supplementary Fig. 5: optoMCP-FUS condensation does not lead to either stress granule formation or changes in global protein translation

(A) Immunofluorescence images of G3BP1 in the optoMCP-FUS expressing U2OS cells with the MBS-PBS labeled tagBFP reporter. (B) (Top) Confocal fluorescence images of OP-Puro signals in the optoMCP-FUS expressing U2OS cells with the MBS-PBS labeled tagBFP reporter. (Bottom) OPP intensity in individual cells for different experimental conditions. Data are mean ± SD. (n = 32–95 cells). ****p < 0.0001. p = 4.32E-29 (Blue light and CHX) and 1.93E-78 (Blue light and No OPP). Source data for panel B are provided in the Source Data file.

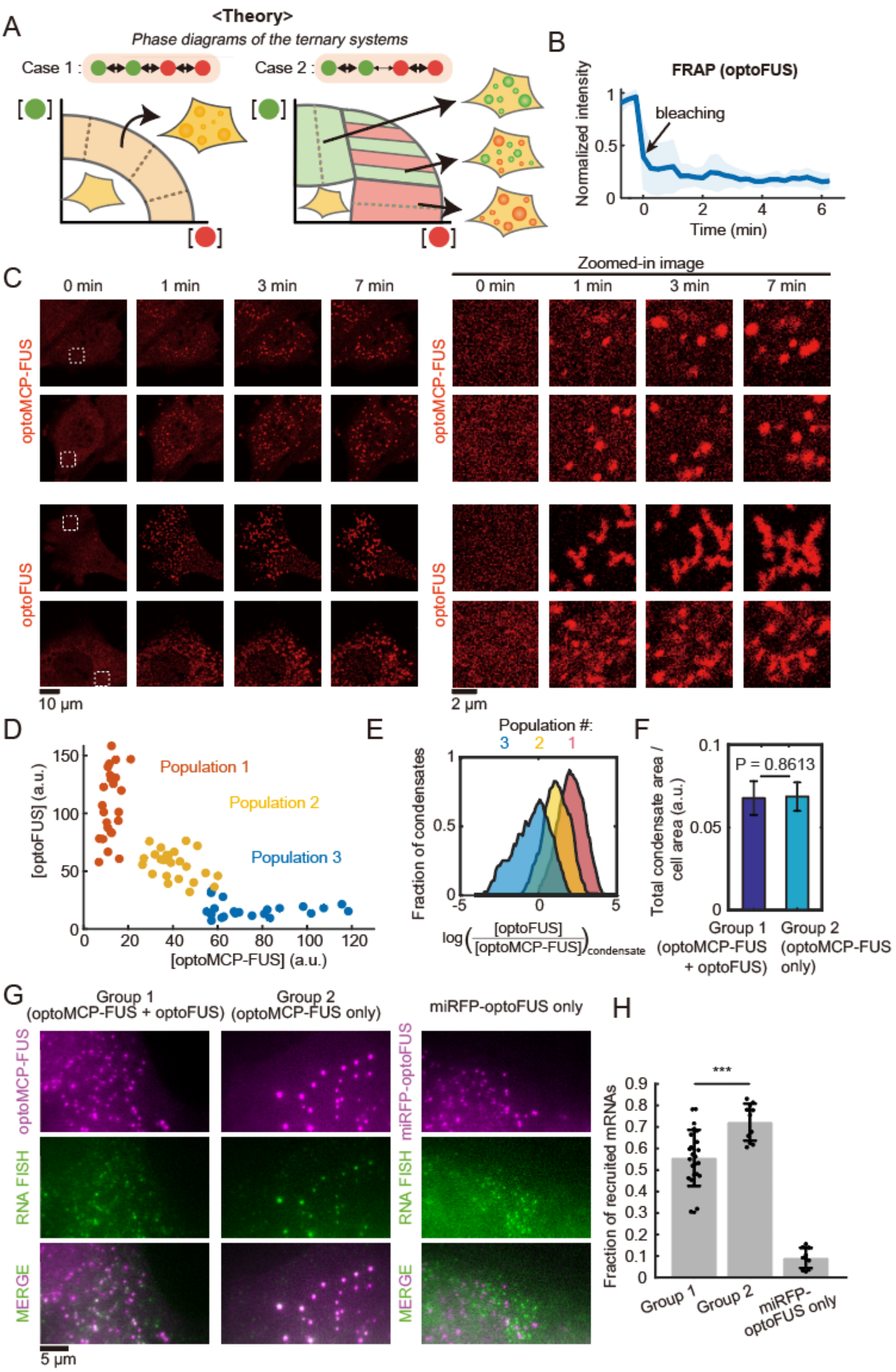

Supplementary Fig. 6: Modulation of condensate material properties through composition controls

(A) Schematic of phase diagrams of ternary systems with two different cases of inter-particle interactions. In both cases, strong homotypic interactions drive phase separation of the same kind

of particles. When homotypic interactions are significantly strong enough to make two coexistence regions intersect in the middle of the phase diagram, two different scenarios are possible. When the strength of heterotypic interactions is comparable to the homotypic ones, a single coexistence region is present (left). If the heterotypic interaction is less favored, two different dense phases, each enriched in the single type of particles, can coexist. (B) FRAP recovery curve for miRFP-optoFUS condensates in U2OS cells. Cells were activated every 15 s to induce phase separation. Data are mean (solid line) ± SD (shaded area). n = 10. (C) Time-lapse confocal images showing phase separation dynamics in MEF cells expressing either optoMCP-FUS or optoFUS. Cells were activated every 15 s. (D) Scatter plot of optoMCP-FUS and optoFUS intensities in individual condensates of two representative U2OS cells from each population in Fig. 4D. (E) Histograms of the intensity ratio of optoFUS to optoMCP-FUS in individual condensates from each U2OS cell population. n = 7 cells for each population. (F) Total condensate areas were measured and divided by cell areas for individual cells in Group 1 and 2 in Fig. 4F. a.u., arbitrary units. Data are mean ± SD. (G) Two-color fluorescence images of fixed U2OS cells, with the MBS-PBS labeled tagBFP reporter, expressing (left) optoMCP-FUS and miRFP-optoFUS or (center) optoMCP-FUS alone or (right) miRFP-optoFUS alone. The MBS-PBS reporter mRNAs, visualized with smFISH (green) and light-activated condensates (magenta), are shown. Images are maximum-intensity projections. (H) Fraction of mRNAs recruited into the light-activated condensates. After 20 min of blue light activation, the recruitment of mRNAs into light-activated condensates was quantified from smFISH data (See Methods). n = 25 cells for Group 1, n = 11 cells for Group 2, and n = 10 cells for miRFP-optoFUS control. ***$p < 0.001$. $p = 0.0005$. Data are mean ± SD. Source data for panel B, D, E, F and H are provided in the Source Data file.

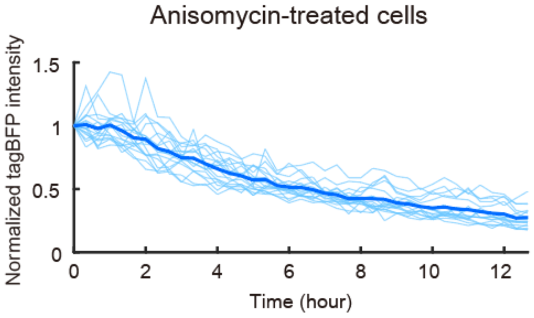

Supplementary Fig. 7: The decay of tagBFP fluorescence upon the treatment of anisomycin

TagBFP fluorescence levels were measured after the treatment of 50 μM anisomycin, a protein synthesis inhibitor, in individual U2OS cells with the tagBFP mRNA reporter. The bold curves are averaged values. n= 17 cells. Source data are provided as a Source Data file.

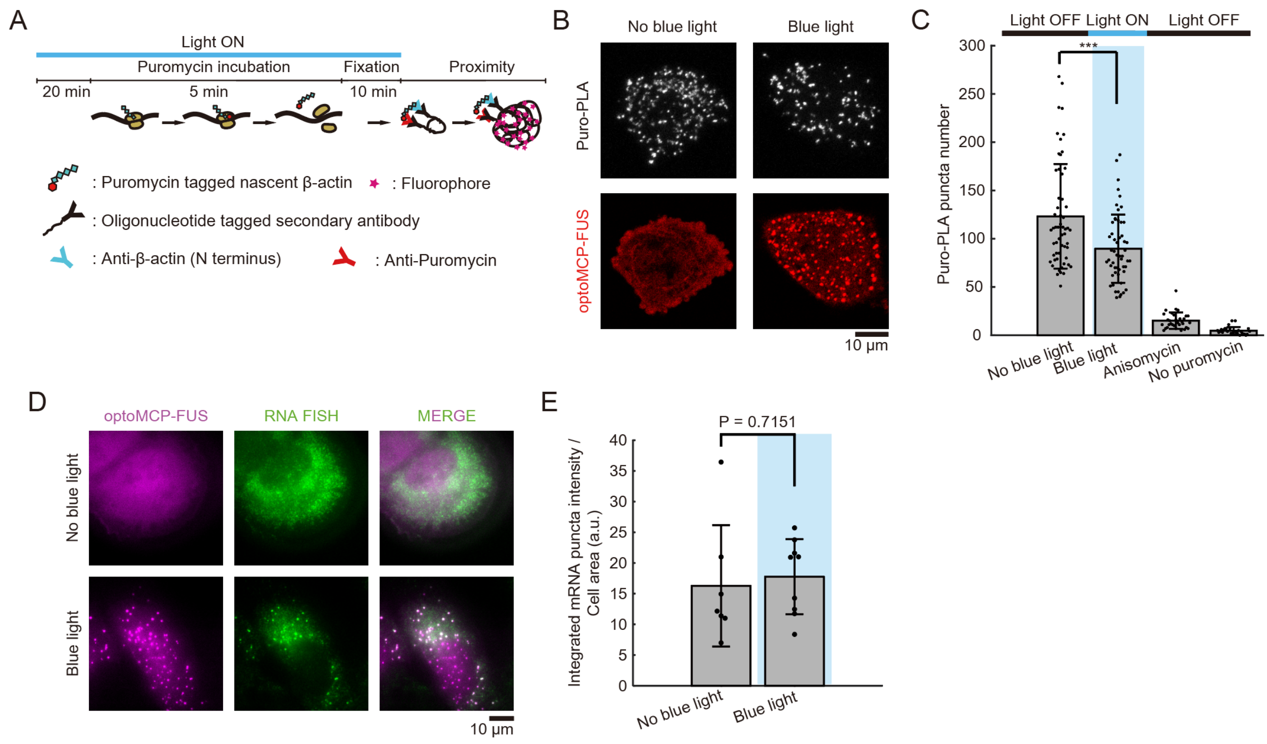

Supplementary Fig. 8: Puro-PLA analysis reveals that condensate-mediated translation repression takes place in short time scales

(A) Schematic of experiments for Puro-PLA assay. Cells were treated with a low concentration (3 μM) of puromycin which tags nascent polypeptides and thereby releasing them from ribosomes. Puromycylated truncated β-actin polypeptides were then recognized by anti-puromycin and anti-β-actin antibodies. Then, the primary antibodies were recognized by secondary antibodies, and the complexes were visualized with rolling-circle amplification. (B) Representative confocal images of cells acquired through the Puro-PLA assay. The optoMCP-FUS expressing MEF cells with the MBS-tagged β-actin gene were used for the Puro-PLA assay in the presence or absence of blue light activation. Images are maximum-intensity projections. (C) The number of Puro-PLA puncta in MBS-KI MEF cells expressing optoMCP-FUS. n = 56 (No blue light), 54 (Blue light), 32 (Anisomycin), and 29 (No puromycin). ***$p < 0.001$. $p = 2.21E-04$. Data are mean ± SD. (D) Two-color fluorescence images of fixed U2OS cells, with the MBS-PBS labeled tagBFP reporter, expressing optoMCP-FUS. The MBS-PBS reporter mRNAs, visualized with smFISH (green) and light-activated condensates (magenta), are shown. (E) Integrated mRNA puncta intensity divided by cell area. After 20 min of blue light activation, MBS-PBS reporter mRNA signals were quantified using smFISH data of U2OS cells expressing optoMCP-FUS (See Methods). n = 7 (no blue light) and 9 (blue light). Data are mean ± SD. Source data for panel C and E are provided in the Source Data file.

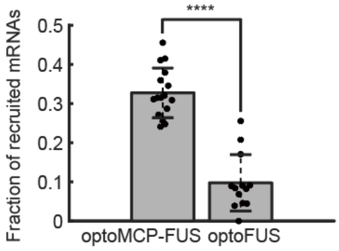

Supplementary Fig. 9: mRNA recruitment into the light-activated condensates in neuronal cells

Fraction of mRNAs recruited into the light-activated condensates in neuronal cells with the MBS tagged β-actin gene expressing optoMCP-FUS or optoFUS. After 20 min of blue light activation, the recruitment of mRNAs into light-activated condensates is quantified from smFISH data (See Methods). Data are mean ± SD. n = 16 (optoMCP-FUS) and 13 (optoFUS). Statistical significance was calculated using Student's two-tailed t-test. ****$p < 0.0001$. $p = 9.17E-10$. Source data are provided as a Source Data file.
