# Supplementary material for: Optogenetic control of mRNA condensation reveals an intimate link between condensate material properties and functions
Source: Nat Commun. 2024 Apr 15;15:3216. doi: 10.1038/s41467-024-47442-x (PMC11018775; doi:10.1038/s41467-024-47442-x)
Supplement: Supplementary file 3 — Description of Additional Supplementary Information [file 41467_2024_47442_MOESM3_ESM.pdf]

Description of Additional Supplementary Files

File Name: Supplementary Data 1
Description: Sequence of the probes used for smFISH and primers used for PCR.

File Name: Supplementary Movie S1
Description: Light activation of the optoMCP-FUS expressing MEF cell with the MBS-tagged β-actin gene. The cell was activated and imaged with the confocal microscope every 2 seconds.

File Name: Supplementary Movie S2
Description: Deactivation of the optoMCP-FUS expressing MEF cell with the MBS-tagged β-actin gene. The cell was imaged with the confocal microscope every 20 seconds.

File Name: Supplementary Movie S3
Description: mRNA imaging through mCherry signals of optoMCP-FUS in the MEF cell with the MBS-tagged β-actin gene. The cell was imaged with the widefield microscope every 155 ms.

File Name: Supplementary Movie S4
Description: Transient interactions between the optoMCP-FUS condensate and the target mRNA. The MEF cell expressing stdPCP-stdGFP and optoMCP-FUS and containing 12x(PBS-MBS)-tagged endogenous c-FOS gene was imaged. The cell is exposed under blue light and imaged every 50 ms. Images were walking-averaged, bleach-corrected, and drift-corrected.

File Name: Supplementary Movie S5
Description: Stable interactions between the optoMCP-FUS condensate and the target mRNA. The MEF cell expressing stdPCP-stdGFP and optoMCP-FUS and containing 12x(PBS-MBS)-tagged endogenous c-FOS gene was imaged. The cell is exposed under blue light and imaged every 50 ms. Images were walking-averaged, bleach-corrected, and drift-corrected.
