# Supplementary material for: Optogenetic control of mRNA condensation reveals an intimate link between condensate material properties and functions
Source: Nat Commun. 2024 Apr 15;15:3216. doi: 10.1038/s41467-024-47442-x (PMC11018775; doi:10.1038/s41467-024-47442-x)
Supplement: Supplementary file 10 — Reporting Summary [file 41467_2024_47442_MOESM10_ESM.pdf]

nature portfolio

Corresponding author(s): Yongdae Shin

Last updated by author(s): Jan 8, 2024

Reporting Summary

Nature Portfolio wishes to improve the reproducibility of the work that we publish. This form provides structure for consistency and transparency in reporting. For further information on Nature Portfolio policies, see our Editorial Policies and the Editorial Policy Checklist.

Statistics

For all statistical analyses, confirm that the following items are present in the figure legend, table legend, main text, or Methods section.

n/a	Confirmed	
☐	☒	The exact sample size (*n*) for each experimental group/condition, given as a discrete number and unit of measurement
☐	☒	A statement on whether measurements were taken from distinct samples or whether the same sample was measured repeatedly
☐	☒	The statistical test(s) used AND whether they are one- or two-sided<br>*Only common tests should be described solely by name; describe more complex techniques in the Methods section.*
☒	☐	A description of all covariates tested
☒	☐	A description of any assumptions or corrections, such as tests of normality and adjustment for multiple comparisons
☐	☒	A full description of the statistical parameters including central tendency (e.g. means) or other basic estimates (e.g. regression coefficient) AND variation (e.g. standard deviation) or associated estimates of uncertainty (e.g. confidence intervals)
☐	☒	For null hypothesis testing, the test statistic (e.g. *F*, *t*, *r*) with confidence intervals, effect sizes, degrees of freedom and *P* value noted<br>*Give P values as exact values whenever suitable.*
☒	☐	For Bayesian analysis, information on the choice of priors and Markov chain Monte Carlo settings
☒	☐	For hierarchical and complex designs, identification of the appropriate level for tests and full reporting of outcomes
☒	☐	Estimates of effect sizes (e.g. Cohen's *d*, Pearson's *r*), indicating how they were calculated

Our web collection on statistics for biologists contains articles on many of the points above.

Software and code

Policy information about availability of computer code

Data collection	Fluorescence images were acquired using either Olympus IX73 inverted microcope, Nikon Eclipse Ti-E inverted wide-field microscope, or a Nikon A1 laser scanning confocal microscope.
Data analysis	The data analysis was performed with Excel (2016-Microsoft 365), ImageJ (version 1.53c-v), and Matlab (R2019b-R2022b).

For manuscripts utilizing custom algorithms or software that are central to the research but not yet described in published literature, software must be made available to editors and reviewers. We strongly encourage code deposition in a community repository (e.g. GitHub). See the Nature Portfolio guidelines for submitting code & software for further information.

Data

Policy information about availability of data

All manuscripts must include a data availability statement. This statement should provide the following information, where applicable:

- Accession codes, unique identifiers, or web links for publicly available datasets
- A description of any restrictions on data availability
- For clinical datasets or third party data, please ensure that the statement adheres to our policy

All relevant data supporting the key findings of this study are available within the paper and its supplementary information files.

Research involving human participants, their data, or biological material

Policy information about studies with human participants or human data. See also policy information about sex, gender (identity/presentation), and sexual orientation and race, ethnicity and racism.

Reporting on sex and gender	N/A, No human participants.
Reporting on race, ethnicity, or other socially relevant groupings	N/A, No human participants.
Population characteristics	N/A, No human participants.
Recruitment	N/A, No human participants.
Ethics oversight	N/A, No human participants.

Note that full information on the approval of the study protocol must also be provided in the manuscript.

Field-specific reporting

Please select the one below that is the best fit for your research. If you are not sure, read the appropriate sections before making your selection.

☒ Life sciences ☐ Behavioural & social sciences ☐ Ecological, evolutionary & environmental sciences

For a reference copy of the document with all sections, see nature.com/documents/nr-reporting-summary-flat.pdf

Life sciences study design

All studies must disclose on these points even when the disclosure is negative.

Sample size	Sample sizes for live cell microscopy were chosen according to commonly used and accepted standards in the field. (PMID : 28041848)
Data exclusions	No data were excluded.
Replication	All experiments were performed independently more than three times. The same pattern was successfully reproduced in all trials.
Randomization	Randomization was not feasible or necessary for this study as it involved live imaging of cells by a single investigator.
Blinding	Since all experiments used in this study did not require group allocations, blinding is not relevant for our study.

Reporting for specific materials, systems and methods

We require information from authors about some types of materials, experimental systems and methods used in many studies. Here, indicate whether each material, system or method listed is relevant to your study. If you are not sure if a list item applies to your research, read the appropriate section before selecting a response.

Materials & experimental systems

n/a	Involved in the study	
☐	☒	Antibodies
☐	☒	Eukaryotic cell lines
☒	☐	Palaeontology and archaeology
☐	☒	Animals and other organisms
☒	☐	Clinical data
☒	☐	Dual use research of concern
☒	☐	Plants

Methods

n/a	Involved in the study	
☒	☐	ChIP-seq
☒	☐	Flow cytometry
☒	☐	MRI-based neuroimaging

Antibodies

Antibodies used	For primary antibodies, Anti-G3BP1 (Abcam, ab56574), Anti-Puromycin (Kerafast, EQ0001) and Anti-Beta-actin (Abcam, ab8227) were used. For PLA step, Donkey anti-Mouse IgG (H+L) (Duolink,DUO92004) and Donkey anti-Rabbit IgG (H+L) (Duolink, DUO92002) were used.

Validation	The quality of the antibodies utilized was verified through tests conducted by the manufacturers or through relevant citations provided on their websites. Further details regarding the validation process are available on the websites of the respective manufacturers.<br>Anti-G3BP1 (Abcam, ab56574) : https://www.abcam.com/products/primary-antibodies/g3bp-antibody-2f3-ab56574<br>Anti-Puromycin (Kerafast, EQ0001) : https://www.kerafast.com/productgroup/190/anti-puromycin-3rh11-antibody<br>Anti-Beta-actin (Abcam, ab8227) : https://www.abcam.com/products/primary-antibodies/beta-actin-antibody-ab8227<br>Anti-Mouse IgG (H+L) (Duolink,DUO92004) : https://www.sigmaaldrich.com/KR/ko/product/sigma/duo92004<br>Anti-Rabbit IgG (H+L) (Duolink, DUO92002) : https://www.sigmaaldrich.com/KR/ko/product/sigma/duo92002

Eukaryotic cell lines

Policy information about cell lines and Sex and Gender in Research

Cell line source(s)	Lenti-X 293T cells were purchased from Takara Bio. U2OS cells were purchased from KCLB (Korean Cell Line Bank).
Authentication	Cell lines used were not authenticated.
Mycoplasma contamination	The cells were not specifically tested mycoplasma contamination.
Commonly misidentified lines<br>(See ICLAC register)	None of the cell lines used were listed in the ICLAC database

Animals and other research organisms

Policy information about studies involving animals; ARRIVE guidelines recommended for reporting animal research, and Sex and Gender in Research

Laboratory animals	The following mouse lines were used:<br>-Wild-type C57BL/6 strain mice<br>-Homozygous ActB-MBS mice<br>Both male and female postnatal day 1 (P1) pups were used for neuron culture experiments.<br>Wild-type C57BL/6 pups were obtained from Koatech (Pyeongtaek-si, Gyeonggi-do, Republic of Korea).
Wild animals	No wild animals were used in this study.
Reporting on sex	Both female and male pubs (postnatal day 1) were used for neuron culture experiments.
Field-collected samples	No field-collected samples were used in this study.
Ethics oversight	All experimental procedures and protocols were conducted with the approval of the Seoul National University (SNU) Institutional Animal Care and Use Committee (IACUC) under license number SNU-191219-1-3.

Note that full information on the approval of the study protocol must also be provided in the manuscript.

Plants

Seed stocks	*Report on the source of all seed stocks or other plant material used. If applicable, state the seed stock centre and catalogue number. If plant specimens were collected from the field, describe the collection location, date and sampling procedures.*
Novel plant genotypes	*Describe the methods by which all novel plant genotypes were produced. This includes those generated by transgenic approaches, gene editing, chemical/radiation-based mutagenesis and hybridization. For transgenic lines, describe the transformation method, the number of independent lines analyzed and the generation upon which experiments were performed. For gene-edited lines, describe the editor used, the endogenous sequence targeted for editing, the targeting guide RNA sequence (if applicable) and how the editor was applied.*
Authentication	*Describe any authentication procedures for each seed stock used or novel genotype generated. Describe any experiments used to assess the effect of a mutation and, where applicable, how potential secondary effects (e.g. second site T-DNA insertions, mosiacism, off-target gene editing) were examined.*
